# The Role of *her4* in Inner Ear Development and Its Relationship with Proneural Genes and Notch Signalling

**DOI:** 10.1371/journal.pone.0109860

**Published:** 2014-10-09

**Authors:** Marija Radosevic, Laura Fargas, Berta Alsina

**Affiliations:** Departament de Ciències Experimentals i de la Salut, Universitat Pompeu Fabra, Parc de Recerca Biomèdica de Barcelona, Barcelona, Spain; Texas A&M University, United States of America

## Abstract

The generation of sensory neurons and hair cells of the inner ear is under tight control. Different members of the *Hairy and Enhancer of Split* genes (HES) are expressed in the inner ear, their full array of functions still not being disclosed. We have previously shown that zebrafish *her9* acts as a patterning gene to restrict otic neurogenesis to an anterior domain. Here, we disclose the role of another her gene, *her4*, a zebrafish ortholog of *Hes5* that is expressed in the neurogenic and sensory domains of the inner ear. The expression of *her4* is highly dynamic and spatiotemporally regulated. We demonstrate by loss of function experiments that in the neurogenic domain *her4* expression is under the regulation of *neurogenin1* (*neurog1*) and the Notch pathway. Moreover, *her4* participates in lateral inhibition during otic neurogenesis since *her4* knockdown results in overproduction of the number of *neurog1* and *deltaB*-positive otic neurons. In contrast, during sensorigenesis *her4* is initially Notch-independent and induced by *atoh1b* in a broad prosensory domain. At later stages *her4* expression becomes Notch-dependent in the future sensory domains but loss of *her4* does not result in hair cell overproduction, suggesting that there other *her* genes can compensate its function.

## Introduction

Distinct proneural genes from the atonal basic helix-loop-helix (bHLH) superfamily control cell fate specification in the inner ear. *Drosophila atonal* gene was first identified in 1993 [1], its mutation resulting in absence of PNS chordotonal organs and photoreceptors [2]. In vertebrate auditory system one of the *atonal* orthologs, *atoh1*, is initially expressed in the sensory epithelium to later restrict in nascent hair cells [3]. Either the loss or overexpression of *atoh1* results in complete absence or ectopic differentiation of hair cells [4]–[6]. In zebrafish, two *atoh1* genes are found in the inner ear, which act in two distinct phases of sensory development [7]. Initially, *atoh1b* appears in a broad prosensory territory to subsequently get restricted to smaller sensory domains. There, *atoh1a* is induced by *atoh1b* to direct the differentiation of hair cells [7]. In all organisms studied, the Notch pathway regulates the production of hair cells and supporting cells via lateral inhibition [8]–[10].

Another member of *atonal* proneural family, *neurog1*, is used to define the otic neurogenic domain where otic progenitors will acquire a neuronal lineage [11], [12]. Again, Notch signalling is key to regulate the final number of otic sensory neurons [8], [9], [13], [14]. The activities of Notch pathway and proneural genes are highly intertwined in many developmental contexts, generally conceived to work in concert. However, a detail mapping of Notch activity throughout inner ear development in correlation with proneural expression is still missing. The lack of good antibodies to detect the cleaved form of Notch Intracellular Domain (NICD) in other species than mouse hampers identifying Notch active cells, with only one report showing NICD localization during cochlear development [15]. Alternatively, expression of HES genes, considered direct targets of Notch pathway has been used to monitor Notch activity. Upon Notch activation *Hes5* is induced and suppresses hair cell fate while promoting supporting cell fate [16]–[18]. Similarly, *Hes5* is expressed complementary to *Delta1* expressing cells in the neurogenic domain [13]. *Her4* is one of the zebrafish orthologs of mammalian *Hes5* and it has been implicated in the control of primary neurogenesis, brain regeneration and neuronal target innervation [19]–[21]. In the CNS *her4* expression depends on Notch, while does not in the trigeminal sensory ganglia [22]. Nothing is known of *her4* in the inner ear, thus we aimed to study its function and regulation during neural and sensory specification. Here we show that zebrafish *her4* is expressed in the neurogenic and sensory domains and requires *neurog1* and *atoh1b* for its expression, respectively. Moreover, *her4* in the neurogenic domain is dependent on Notch, while initial broad induction of *her4* in the presumptive sensory domain does not require Notch but *atoh1b*. However, later on Notch restricts *her4* expression to the future sensory maculae. Furthermore, we show that loss of *her4* mediates lateral inhibition during neurogenesis, but not during sensorigenesis.

## Materials and Methods

### Fish maintenance and zebrafish stains

Zebrafish were maintained at the PRBB Animal Facility under standard conditions. The zebrafish protocols followed the guidelines and were approved by the IACUC, Comité Ético de Experimentación Animal-Parc de Recerca Biomèdica de Barcelona (CEEA-PRBB); approved number JIB-08-1098P2. The zebrafish strains used were the following: wild-type (AB), mib^ta52b^ [23] and *neurog1^hi^* ^1059^ [24], [25]. We also used the transgenic line Tg(her4:EGFP)^y83^ [22]. A 3.4 kb 5′-flanking region of *her4* DNA containing 22 bp of 5′ UTR sequence of her4 cDNA is controlling EGFP expression. Homozygous mutant *mib* and *neurog1* embryos were obtained by pairwise mating of heterozygous adult carriers. *mib* descendant embryos were genotyped after *in situ* hybridization. Embryos were developed in an incubator at 28.5°C in system water containing methylene blue and staged after counting somite number. Embryonic stages are given as hours post-fertilization (hpf) at 28.5°C [26].

### Whole mount *in situ* hybridization

Synthesis of antisense RNA and whole-mount *in situ* hybridization were performed as previously described [27]. Probes used were as follows: *atoh1b* and *atoh1a* [7], *neurog1* [28], *her4* [29] and *deltaB* [8]. Embryos were isolated at desired developmental stages essentially as described [26]. Dechorionated zebrafish embryos were fixed in 4% paraformaldehyde (PFA) overnight at 4°C and dehydrated in methanol series, rehydrated again and permeabilized with 10 mg/ml proteinase K (Sigma) at RT for 5–10 min depending on their stage. Digoxigenin-labeled probes were hybridized overnight at 70°C, detected using anti-digoxigenin-AP antibody at 1∶2000 dilution (Roche) and developed with NBT/BCIP (Roche). Embryos were post-fixed overnight in 4% PFA and used for imaging either mounted in 100% glycerol or cryoprotected in 15% sucrose and embedded in 7.5%gelatine/15%sucrose. Blocks were frozen in 2-Methylbutane (Sigma) for tissue preservation and cryosectioned at 20 µm on a Leica CM 1510-1 cryostat.

### Antisense morpholinos (MO)

MOs were obtained from Gene Tools. Embryos were injected at 1-cell stage. The *her4*-MO [21] was designed to block the translation of *her4* mRNA transcript with sequence: 5′-ATT GCT GTG TGT CTT GTG TTC AGT T-3′. *Her4*-MO was injected at concentration 0.025 mM and its efficiency was assessed by the specific loss of GFP signal from the Tg(her4:EGFP)^y83^ transgenic line [22]. The *atoh1b*-MO [7] was injected at concentration 5 µg/µl and its sequence is 5′-TCA TTG CTT GTG TAG AAA TGC ATA T-3′.

### SU5402 inhibitor treatment

Dechorionated zebrafish embryos were incubated with 50 µM SU5402 (Calbiochem, 572630), a potent pharmacological inhibitor of Fgf signalling. Incubations were done at 28.5°C, starting at 10 hpf until the sacrifice of the animals at 16 hpf. The final solution was done in E3 medium from the 5 mM SU5402 stock solution (kept at −20°C in DMSO). For control treatments, embryos were incubated in an equivalent concentration of dimethyl sulfoxide (DMSO, Sigma).

### Image acquisition

Pictures were acquired in a Leica DRM microscope or in Leica MZFLIII stereomicroscope using a Leica DFC300 FX camera and the Leica IM50 software. Adobe Photoshop 7.0.1 software was used for photograph editing.

## Results

### Spatiotemporal dynamics of *her4* expression in relation to proneural genes

**Figure 1:**
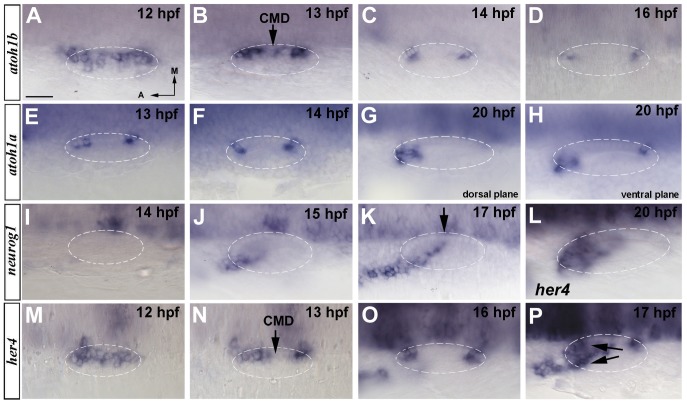
Spatiotemporal expression of proneural genes at early stages of inner ear development. (**A–D**) In situ hybridization in wild-type embryos for *atoh1b. atoh1b* is expressed in a large medial domain adjacent to the hindbrain at 12 hpf (A) to then progressively restrict to two patches that correspond to the future anterior and posterior prosensory domains (B–D). Between 13 hpf and 14 hpf *atoh1b* is downregulated at the central medial domain (CMD) (arrow in B). (**E–H**) In situ hybridization in wild-type embryos for *atoh1a. atoh1a* is expressed in the anterior and posterior sensory domains by 13 hpf and 14 hpf (E, F). At 20 hpf the expression in the anterior domain increases (G) and just two positive cells remain in the posterior (H). (**I–K**) In situ hybridization in wild-type embryos for *neurog1*. Expression of *neurog1* in the inner ear starts at 15 hpf (J) and it is extended from an anterolateral position to a posteromedial domain (K). *neurog1* progressively invades the CMD (arrow in K). (**L–P**) In situ hybridization in wild-type embryos for *her4. her4* expression is found at 12 hpf (M) to become restricted to the future sensory maculae by 13 hpf (N). At 16 hpf *her4* presents higher expression levels (O) and at 17 hpf, the expression of *her4* appears in the neurogenic domain being a sum of the expressions from both domains (arrows in P). By 20 hpf the expression only remains at the anterior sensory domain (L). Dorsal views; anterior to the left, medial to the top. Dashed circles delineate otic vesicles. All images are at same magnification. Scale bar: 25 µm.

The family of *her* genes in zebrafish, orthologous to mammalian Hes genes, consists of 21 genes based on data published on Ensembl (Zv9). We analysed the expression of 10 *her* genes during otic development and only *her6*, *her4* and *her9* were found to be expressed in the otic vesicle from 12–14 hpf to 24 hpf (data not shown). We have previously demonstrated a role for *her9* during otic patterning in restricting neurogenesis to the anterior domain, downstream of retinoic acid (RA) signalling [30] and here concentrated on *her4* gene. Previous studies have analysed the development of the neurogenic and sensory territories separately, however since both domains develop simultaneously, a complete picture of the development of both territories should be taken into account. Moreover, the sequential phases of Notch activation is still not well defined. Consequently, we have generated a precise map of the expression of *her4* at early stages of neuro- and sensorigenesis together with the expression of *atoh1b*, *atoh1a* and *neurog1*. *her4* expression is first observed at 12 hpf throughout a broad band of cells just adjacent to the hindbrain. The pattern at this stage is strikingly similar to the one from *atoh1b* (compare Figure 1M with Figure 1A). By 13 hpf, both genes start to become down-regulated from the Central Medial Domain (CMD) of the otic placode and higher expression is observed in anterior and posterior patches (Figure 1N and Figure 1B, arrow pointing to CMD; [7]). At 16 hpf, *her4* is only detected at the future anterior (utricular) and posterior (saccular) sensory maculae with higher levels of expression than *atoh1b* (compare Figure 1O with Figure 1C–D). *atoh1a* is expressed solely at the future anterior and posterior sensory domains from 13 hpf onwards (Figure 1E–H and [7], [8]). At 20 hpf, the number of cells expressing *atoh1a* in the anterior macula increases, while only two cells remain expressing *atoh1a* at the posterior macula (Figure 1G and H) (as a result of the spherical shape of the otic vesicle, the posterior macula expressing *atoh1a* is not visible at dorsal planes but only in ventral planes).

Together with the activation of *atoh1*, another proneural gene, *neurog1*, is induced in the inner ear. In zebrafish, in contrast to what happens in other vertebrate species such as chick and mouse, sensory specification by *atoh1* genes takes place before neuronal specification [7], [9], [31], [32]. The expression of *neurog1* is first detected at 15 hpf in the most anterolateral cells of the otic placode to progress overtime to more medial positions (Figure 1I and K, [30]). The expression of *neurog1* lags the first appearance of *atoh1b* by three hours. At 17 hpf, *her4* expression in the neurogenic domain appears, being the expression in the otic vesicle a sum of the expressions from the sensory and the neurogenic domains (Figure 1P, arrows pointing to the sensory and neurogenic expression of *her4*). Note that *neurog1* expression begins in the neurogenic domain at 15 hpf but *her4* expression is not initiated there until two hours later (compare Figure 1J, 1O and 1P). Later on, from 20 hpf *her4* expression in the sensory domains remains only apparent in the anterior macula (Figure 1L). From the described expression patterns over time, we can infer that *her4* is transcribed in both the neurogenic and sensory territories, with dynamics highly similar to those of *atoh1b* and *neurog1*.

### Complex and dynamic regulation of *her4* by Notch in the inner ear

**Figure 2:**
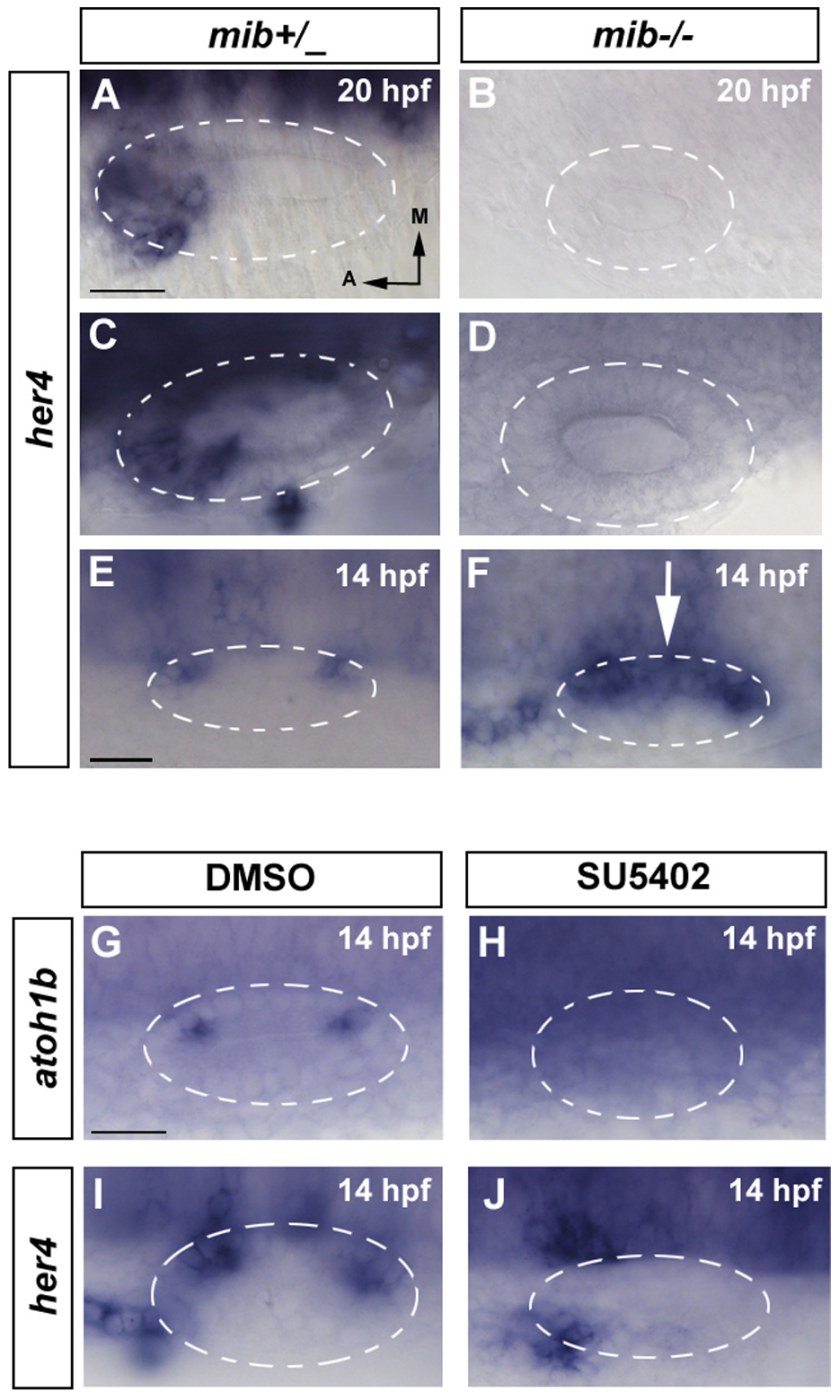
*her4* is regulated by Notch in the neurogenic domain but only partially in the sensory domain. (**A–F**) In situ hybridization for *her4* expression in control and *mib-/-* embryos. (**A, C, E**) *her4* expression in wild-type embryos. At 14 hpf *her4* expression is restricted to the two sensory patches (E). At 20 hpf and 24 hpf *her4* expression is detected in the sensory and neurogenic domains (A, C). (**B, D, F**) *her4* expression in *mib-/-* embryos. At 14 hpf *her4* expression is detected in the anterior and posterior sensory domains, but it is not repressed in the CMD (white arrow in F). At 20 hpf and 24 hpf its expression in the sensory and neurogenic domains is completely abolished (B, D). (**G–J**) In situ hybridization for *atoh1b* and *her4* at 14 hpf in DMSO and SU5402 treated embryos. *atoh1b* (G, H) and *her4* expression (I–J) expression is completely lost in embryos treated with SU5402 (H,J) compared with control embryos treated with DMSO (G,I). Dorsal views; anterior to the left, medial to the top. Dashed circles delineate otic vesicles. Images A–D, E–F and G–J are at same magnification. All scale bars: 25 µm.

In many cases, HES members are activated by Notch [19]. However, this is not a strict rule since in some cases BMP, Wnt or RA pathways but not Notch regulate *her* transcription [30], [33], [34]. Therefore, to test whether *her4* expression is regulated by Notch pathway in the sensory and neurogenic domains, we have analysed the effect of Notch inhibition in *mib^ta52b^* mutant embryos that lack the specific Notch ubiquitin ligase necessary for Delta endocytosis [23]. In *mib^ta52b^* embryos, *deltaB* and *deltaD* are upregulated and lateral inhibition is impaired causing overproduction of hair cells at the expense of supporting cells [8]. When looking at what happens in the neurogenic domain, we found that *her4* expression is abolished in *mib* mutant embryos of 20 hpf and 24 hpf (*mib* genotyped; n = 4/4) compared to heterozygous embryos that retain *her4* expression (Figure 2A–D; n = 3/3). Similarly, *her4* expression is also completely absent from the sensory maculae at 20 hpf and 24 hpf in *mib^ta52b^* embryos (Figure 2A–D), suggesting that at these stages *her4* expression depends on Notch in both, neurogenic and sensory domains.

It has been shown that the initial broad expression of *atoh1b* is set up independently of Notch activity but subsequently Notch represses *atoh1b* in the CMD at 12–13 hpf [7]. In consequence, Notch inhibition results in contiguous band of *atoh1b* expression and ectopic hair cells in the CMD [7], [8]. This led us to ask how Notch regulates *her4* expression at this time frame and whether her4 is the repressor of *atoh1b* in the CMD. In contrast to what happens at 20 hpf, *her4* expression is not abolished in *mib^ta52b^* at 14 hpf in any of the embryos but strong *her4* expression in the anterior and posterior maculae is evident and accompanied by an increase of expression in the CMD (Figure 2F, arrow pointing the CMD; n = 3/3). As mentioned, this phenotype highly resembles the phenotype of *atoh1b* expression in *mib^ta52b^* embryos. Since her4 is induced in the CMD in *mib^ta52b^*, instead of down-regulated, *her4* cannot be the *atoh1b* repressor in this region.

Since initial *atoh1b* expression has been shown to depend on Fgf signalling in the inner ear [7], next we decided to test whether blocking Fgf signalling also affects the induction of *her4*. Indeed, *her4* expression is inhibited when embryos were treated pharmacologically with 50 µM SU5402 from 9 hpf until 14 hpf (n = 5/5) as well as *atoh1b* (n = 5/5) confirming that both have similar regulatory requirements (Figure 2G–J).

Taken together, Notch induces *her4* in the neurogenic domain, while the regulation of *her4* by Notch in the sensory domain is complex and changes over time. In a first phase *her4* is set up by Fgf, but not by Notch signalling. Then, Notch down-regulates *her4* expression in the CMD and finally maintains its expression in the future sensory maculae. These experiments indicate that *her4* is not induced by Notch in the CMD to repress *atoh1b*.

### *atoh1b* is upstream of *her4* in the prosensory territory

Since *her4* and *atoh1b* expression profiles over time are practically indistinguishable and also share the same pattern of regulation by Notch, one can formulate distinct reasoning. On one hand, *her4* and *atoh1b* are under the same regulatory pathways or on the other hand, *atoh1b* and *her4* belong to the same genetic pathway, either *atoh1b* regulating *her4* or viceversa.

**Figure 3:**
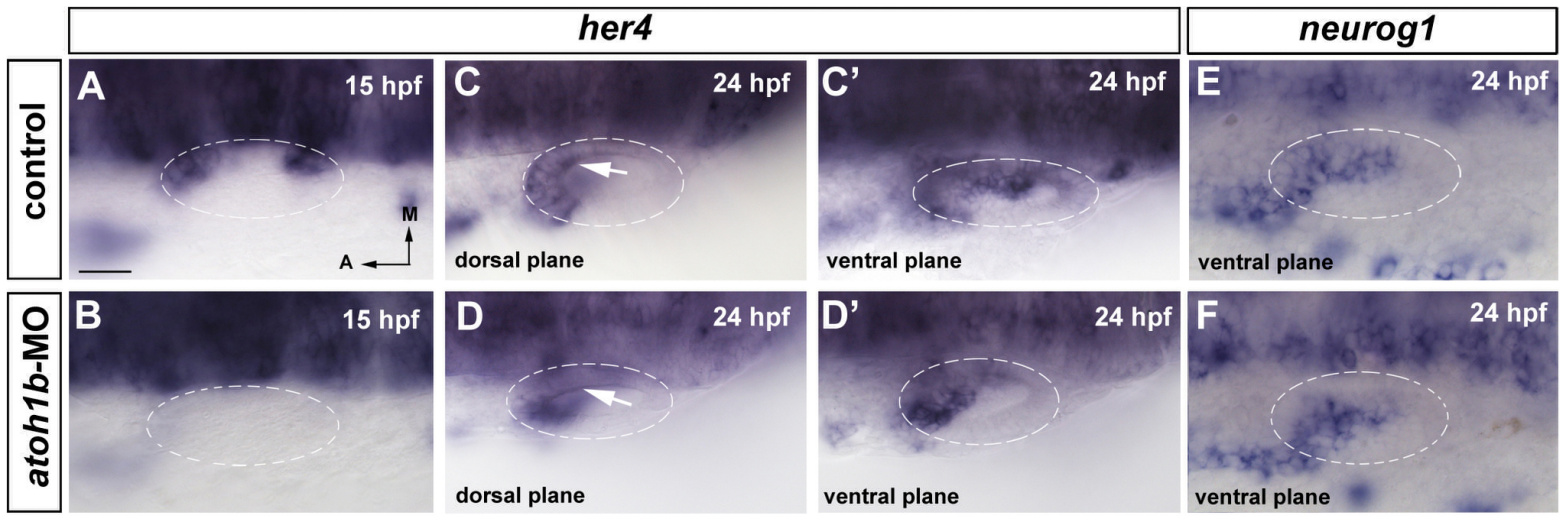
*her4* expression requires *atoh1b* in the sensory domain. (**A–D'**) In situ hybridization for *her4* expression in wild-type and *atoh1b*-MO injected embryos. (**A, B**) In 15 hpf control embryos, *her4* expression is detected in the future anterior and posterior sensory domains (A), while in morphant embryos *her4* expression is lost (B). (**C, D**) At 24 hpf *her4* expression is also lost in the sensory territory in morphant embryos (arrow in D) compared with controls (arrow in C). (**C', D'**) The expression in the neurogenic domain is not affected in morphant embryos. (**E, F**) In situ hybridization for *neurog1* expression in wild-type (E) and *atoh1b*-MO injected (F) embryos. *neurog1* expression in *atoh1b* morphant embryos is not affected. Dorsal views; anterior to the left, medial to the top. Dashed circles delineate otic vesicles. All images are at same magnification. Scale bar: 25 µm.

In order to distinguish between these possibilities, we have blocked *atoh1b* activity by injecting *atoh1b*-morpholino (*atoh1b*-MO) to assess the possible regulation of *her4* by *atoh1b*. *her4* expression precedes the onset of *atoh1a* in the otic placode, thus we reasoned that *atoh1b* instead of *atoh1a* was the best candidate for testing the upstream regulation of *her4*. Indeed, in *atoh1b*-MO injected embryos, expression of *her4* is abrogated in the anterior and posterior sensory epithelia at 15 hpf (Figure 3B; n = 5/5) and no *her4* expression is present in the otic placode, as *her4* is not yet induced in the neurogenic domain. Interestingly, neither precocious nor ectopic expression of *her4* in the neurogenic domain is found. In control conditions, *her4* expression is only present in the future sensory maculae (Figure 3A; n = 5/5). At 24 hpf, again, expression of *her4* is abolished from the sensory territory in *atoh1b* morphant embryos (compare arrow in Figure 3C and D; n = 6/6) but *her4* expression in the neurogenic domain remains intact (compare Figure 3C' with Figure 3D'). The expression of *her4* in the neurogenic domain did not seem to change significantly after blocking *atoh1b*, having into account that there is some intrinsic otic vesicle to otic vesicle variability in the shape and extension of the neurogenic domain. To further confirm this result and analyze a possible cross-talk between *atoh1b* and *neurog1*, we checked for the extent of *neurog1* expression in *atoh1b*-MO injected embryos. Again, no expansion of *neurog1* in the sensory domain was revealed in morphant embryos compared with controls (Figure 3E-F; n = 4 for *atoh1b*-MO and n = 4 for controls).

In summary, *her4* is a target of *atoh1b* in the sensory domain and depletion of *atoh1b* function has no effect on *her4* neither on *neurog1* expression in the neurogenic domain.

### *Neurog1* regulates *her4* expression in the neurogenic domain

**Figure 4:**
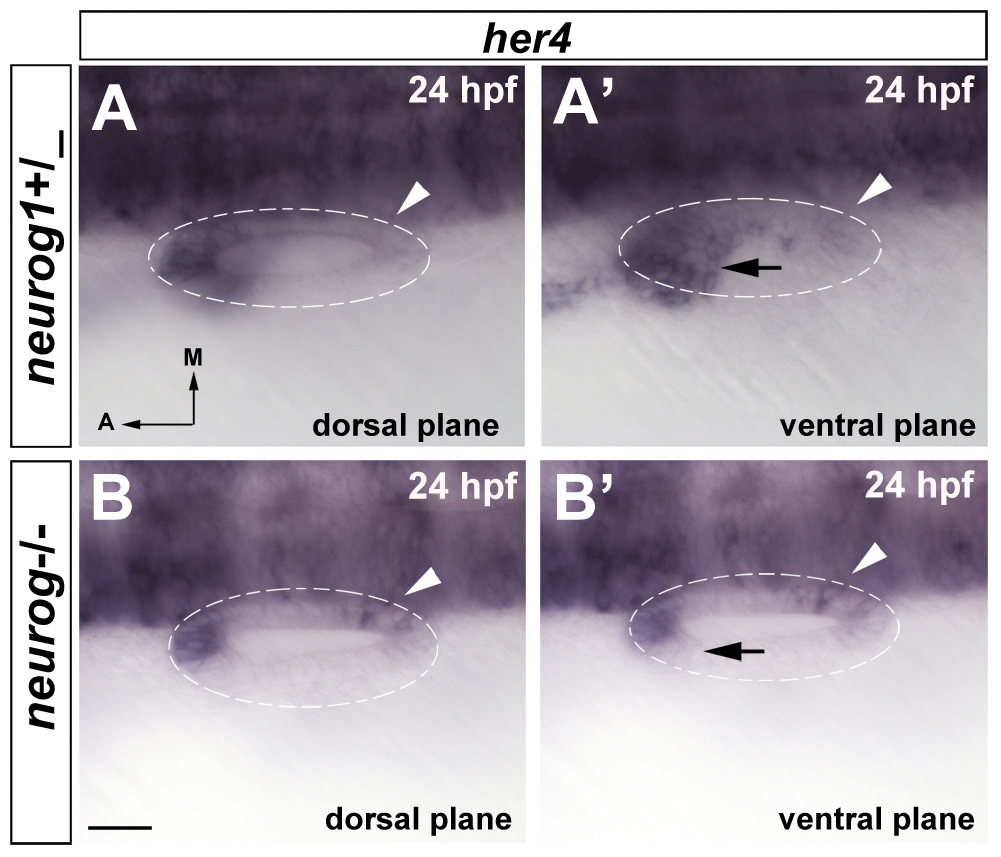
*neurog1* is required to induce *her4* expression in the neurogenic domain. (**A, A'**) In situ hybridization for *her4* expression in wild-type embryos. *her4* expression is detected in both, the anterior sensory domain and the neurogenic domain (arrow A'), but not in the posterior sensory domain (arrowheads A, A'). (**B–B'**) In situ hybridization for *her4* expression in *neurog1-/-* embryos. *Her4* is not expressed in the neurogenic domain (arrow B') and *her4* expression in the posterior sensory domain is detected (arrowheads B, B'). Dashed circles delineate otic vesicles. All images are at same magnification. Scale bar: 25 µm.

We next assessed the genetic relationship of *her4* with the proneural gene *neurog1* by using the *neurog1^hi1059^* mutant line [24]. In *neurog1* ^-/-^ mutants obtained from heterozygotic crosses of *neurog1^hi1059^* adults, *her4* is not expressed in the neurogenic domain at 24 hpf (black arrow in Figure 4B' and Figure 4A'; n = 7/21), whereas expression in the sensory domain is not abolished. Interestingly, the expression of *her4* in wild-type embryos is only detected in the future anterior sensory macula (Figure 4A), whereas in *neurog1* ^-/-^ mutant embryos it is also detectable in the posterior patch (arrowhead in Figure 4B and B'; n = 5/7). This suggests that either down-regulation in the posterior patch does not take place in *neurog1* ^-/-^ mutant embryos or alternatively reactivation of *her4* in the future posterior patch occurs. Therefore, when neurogenesis is impaired in the inner ear from lack of *neurog1, her4* expression in the posterior sensory patch is altered, suggesting a cross-talk in this territory between the different proneural genes. Recently it has been reported that absence of *neurog1* activity expands the posterior sensory patch but not the anterior patch [35], which would be in agreement with our results showing increased *her4* expression at the posterior patch in *neurog1* mutants.

Altogether, our experiments show that *her4* expression is dependent on *atoh1b* and *neurog1* in the sensory and neurogenic domains respectively. Moreover, we corroborate previous studies indicating that *neurog1* influences the development of the posterior macula and we show that *atoh1b* does not influence *neurog1* expression.

### Inhibition of *her4* increases the number of neuronal fated cells but not the number of hair cells

**Figure 5:**
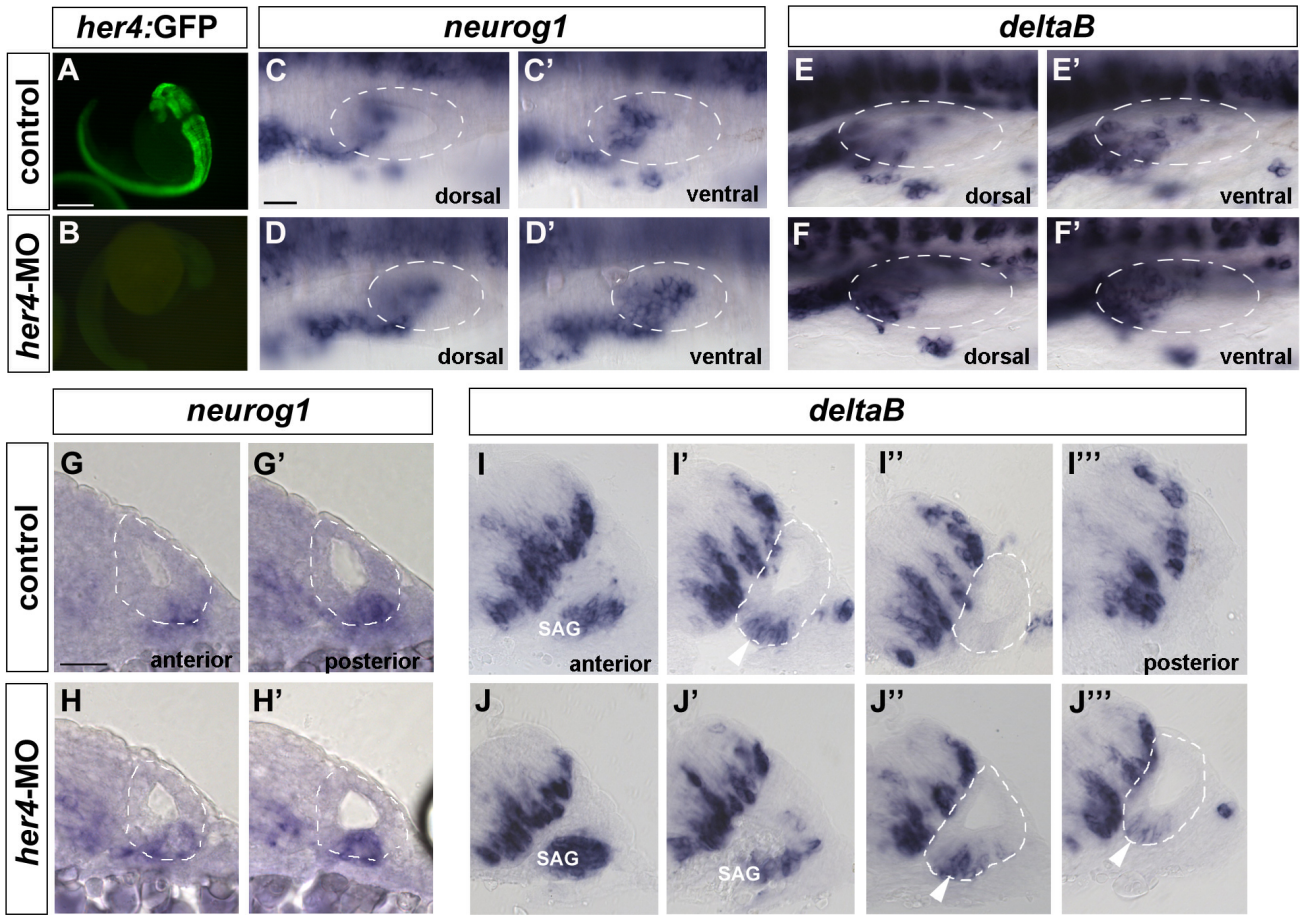
Increased density of *neurog1* and *deltaB*-positive cells in the neurogenic domain after *her4* blockade. (**A, B**) GFP expression in Tg(*her4*:GFP) line. In *her4*-MO injected embryos GFP expression is completely lost (B) compared to controls (A). (**C–D'**) Dorsal views of 24 hpf control (C, C') and *her4*-MO injected (D, D') embryos stained by in situ hybridization for *neurog1*. (D, D') *neurog1* expression in *her4* morphant embryos is increased in the ventral plane (D') compared to controls (C'). (**E–F'**) Dorsal views of 27 hpf control (E, E') and *her4*-MO injected (F, F') embryos stained by in situ hybridization for *deltaB*. (F, F') *deltaB* expression in *her4* morphant embryos is increased in the dorsal and ventral plane (F, F') compared to controls (E, E'). (**G–H'**) Sequential transversal sections; medial to the left, dorsal to the top of 24 hpf control (G, G') and *her4*-MO injected (H, H') embryos stained by in situ hybridization for *neurog1*. In morphant embryos an increased number of cells in the otic epithelium stained for *neurog1* is observed compared to controls. (**I–J'''**) Sequential transversal sections; medial to the left, dorsal to the top of 27 hpf control (I–I''') and *her4*-MO injected (J, J''') embryos stained by in situ hybridization for *deltaB*. In morphant embryos the number of *deltaB*-positive cells in the otic epithelium (compare J'' with I') is increased and also the size of the SAG (J, J'). In control embryos SAG is only present in one section (I). Arrowheads point to epithelial neuroblasts. Dashed circles delineate otic vesicles. Scale bar in A and B: 500 µm, Scale bar in C-F': 25 µm, Scale bar G-J''': 25 µm.

In order to evaluate the role of *her4* during inner ear development, we have blocked *her4* function by injecting *her4*-MO at 1-cell stage embryos. The efficacy of *her4*-MO was assessed in the Tg(*her4*:EGFP) line [22]. In this line, a 3,4 kb region upstream of the her4 start site containing also 22 bpf of the her4 5′UTR is cloned together with EGFP. *Her4*-MO sequence binds specifically to the 5′UTR affecting EGFP translation. In control embryos, strong EGFP is visible in the neural tube, while in 0.025 mM *her4*-MO injected embryos EGFP expression is completely abolished (Figure 5A–B). Next, we have analysed the effects of *her4* on neurogenesis by analysing the expression of *neurog1* and *deltaB* in 24 hpf and 27 hpf *her4* morphant embryos, respectively. Increase of *neurog1* expression is observed in morphant embryos in dorsal and ventral planes compared to controls (Figure 5C–D'; n = 10/18 for *her4*-MO and n = 0/10 for controls). In cross-sections, an expansion of *neurog1* expression is also observed (Figure 5G–H').

During lateral inhibition, cells adopting a primary fate inhibit their neighbours from acquiring the same fate. These cells express ligands of the delta/serrate family to activate the Notch pathway in the adjacent cells. One of the results of Notch activity is the down-regulation of the expression of the delta/serrate ligands, thereby amplifying the differences between neighbouring cells (reviewed in [36], [37]). Several *delta* genes are expressed in the zebrafish inner ear [8], many of them found in the sensory domains. Since delt*aB* is highly expressed in neuronal cells, we chose this ligand to study the effects of loss of *her4* in neuronal differentiation as a possible mediator of Notch signalling during lateral inhibition. When analysing the expression of *deltaB*, increased number of *deltaB*-positive cells was found when analysed in flat-mount images (Figure 5E–F') and in transverse sections (Figure 5I–J'''). In morphant embryos the statoacoustic ganglion (SAG) is bigger, present in two consecutive transverse sections (Figure 5J–J'), compared to one in control embryos (Figure 5I). At the level of the otic epithelium, increased number of neuroblasts was also visible (compare arrowheads in Figure 5J'' and J''' in morphant embryos to Figure 5I' in control embryos), accompanied with higher density of *deltaB*-positive cells in the neural tube. This is in accordance with a role of *her4* in the suppression of a *deltaB* during the selection of neuronal cells upon Notch activation. The results suggest that *her4* regulates *deltaB*, but does not feedback on *neurog1*.

**Figure 6:**
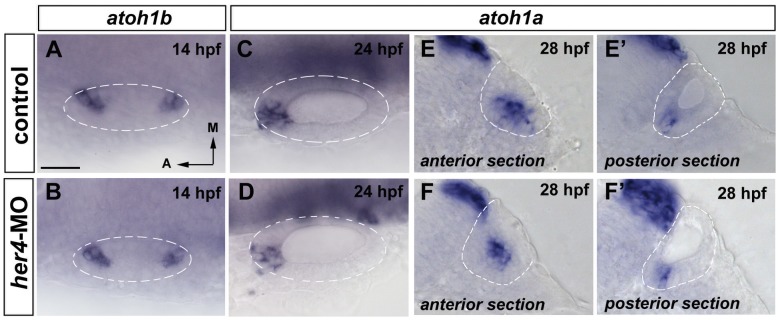
Blockade of *her4* has no increase on *atoh1b* and *atoh1a* expression. (**A–B**) Dorsal views of 14 hpf control (A) and *her4*-MO injected (B) embryos stained by in situ hybridization for *atoh1b*. *atoh1b* expression is not affected in the prosensory domains in *her4*-MO injected embryos. (**C–D**) Dorsal views of 24 hpf control (C) and *her4*-MO injected (D) embryos stained by in situ hybridization for *atoh1a*. *atoh1a* expression decreases in *her4*-MO injected embryos. (**E–F'**) Transversal sections anterior (E, F) and posterior sections (E', F') of 28 hpf control (E, E') and *her4*-MO injected (F, F') embryos stained by in situ hybridization for *atoh1a*. *atoh1a* expression is as wild-type in *her4*-MO injected embryos. Dashed circles delineate otic vesicles. All images are at same magnification. Scale bar: 25 µm.

We could expect a similar role of *her4* in negatively regulating the number of differentiating hair cells in the prosensory domains. First, we have analysed the effects on *atoh1b* to know whether *her4* can control its own main regulator. Interestingly, no effect on the expression of *atoh1b* in morphant embryos was observed at 14 hpf (Figure 6A–B; n = 9/9 for *her4*-MO and n = 4/4 for controls). As we have previously shown that *atoh1b* is upstream of *her4*, this result indicates that, as observed for *neurog1*, *her4* does not feedback on *atoh1b*.

We have seen above that at later stages *her4* becomes dependent of Notch, most probably participating in Notch-mediated lateral inhibition and repressing the differentiation of sensory progenitors to hair cells. If this hypothesis is true, we expected to find increased numbers of hair cells in *her4*-MO embryos. Intriguingly, the number of cells expressing *atoh1a* at 24 hpf and 28 hpf, as a readout of committed hair cells did not increase in *her4*-MO (Figure 6C-F) and was similar to controls. In few injected embryos, we found a reduction in the levels of *atoh1a* (n = 5/13).

## Discussion

Here we have explored the role of *her4* in inner ear development and its relationship with proneural genes and Notch signalling. In the neurogenic domain *her4* and *neurog1* expressions are correlated spatially, with a temporal delay between *neurog1* induction and *her4*, suggesting an intermediate step. The fact that in the neurogenic domain *her4* expression depends on Notch, positions Notch as the intermediary pathway that activates *her4* downstream of *neurog1*. Moreover, depletion of *her4* leads to an increase in the population of neurons. This is similar to what was previously reported for *her4* role in primary neurogenesis [19]. Neither the loss of function of *her4* nor *Hes5* has already been analysed directly in inner ear neurogenesis (all studies being focused on hair cell development). The data demonstrate for the first time that in the inner ear, as in the CNS, *her4* participates in Notch-mediated lateral inhibition to control the final number of neuronal cells.

But what happens in the sensory domain? There, *her4* is regulated differently. In the presumptive sensory territory *her4* expression is highly dynamic and identical to *atoh1b*. It initially encompasses a broad medial territory of the otic placode to progressively restrict to the future anterior and posterior maculae. Initial *her4* expression requires *atoh1b* and Fgf signalling but not Notch, indicating that it cannot be assumed that *her4* is always regulated by Notch. Nonetheless, in an intermediate developmental period, in the CMD Notch regulates negatively but not positively *her4* in the CMD. Our work thus shows that *her4* is not the downstream target of Notch to repress *atoh1b* expression. *her6*, another member of her repressors, cannot perform this role since is not expressed in the CMD at 12.5–13 hpf (Figure S1). Thus, it still remains elusive how Notch represses *atoh1b* in the CMD to obtain two segregated the sensory patches.

Notch, in addition to *her4*, also down-regulates *atoh1b* in the CMD [7]. Since *her4* expression depends on *atoh1b*, we propose that the effect of Notch on *her4* is most probably mediated by *atoh1b*. However, we cannot exclude that Notch inhibits *her4* directly in the CMD in parallel to *atoh1b*. At later stages, *her4* persists at the sensory maculae requiring Notch-activity. Interestingly, by 16 hpf, *her4* expression levels appear higher than *atoh1b*, suggesting that from this period onwards, *her4* expression can be maintained independently of *atoh1b*. This coincides with the period of *atoh1a* activation and probably also Notch pathway. Thus, we propose that by 16 hpf, *her4* regulation changes from a direct regulation by the proneural *atoh1b* to a regulation by *atoh1a* and Notch.

The complex spatiotemporal regulation of *her4* expression suggests multiple cis-regulatory regions controlling *her4* transcription. Yet no data exists on the regulatory regions of this locus. The promoter of mouse *Hes5* has been characterized [38] and, in chick, labels cells responding to Notch and undergoing lateral inhibition during hair cell formation [39]. Here we show evidences for a Notch independent regulation of *her4*, not been described yet in chick and mouse. This might be due to a lack of data at early developmental times or to a divergence in the regulatory mechanisms from anamniote to amniote animals.

The role of Notch in hair cell development has been widely studied. The best known role of Notch in hair cell development is in the process of lateral inhibition, in which cells activated by Notch activate target genes and suppress hair cell fate versus supporting cell fate [8], [10], [40]–[42]. In agreement with a putative role of *her4* downstream of Notch activity during sensorigenesis, we expected to observe an increase in the number of hair cells after *her4* knockdown. However, we failed to observe increased numbers of *atoh1a* expressing cells. This is not due to inefficacy of *her4*-MO since it was tested in Tg(*her4*:EGFP)^y83^ embryos and also it resulted in expansion of *deltaB* expression. One of the most plausible explanations is that contrary to what happens in the neurogenic domain, other *her* genes compensate for *her4* loss in the sensory domain. *Her6*, a *Hes1* ortholog, is expressed exclusively in the sensory domain already from 12.5 hpf (Fig. S1) could act redundantly with *her4*. The presence of *her6* in the sensory patches but not in neurogenic domain could explain why depletion of *her4* has a stronger effect in the latter. This is in agreement with data in mouse, where Hes and Hey genes cooperate in hair cell development and a graded increase in hair cell number was related to a reduction in Hes/Hey dosage, being the highest increase in compound mutants for *Hes1*, *Hes5* and *Hey1* [18].

In chick, the transcription of *atoh1* lags by almost 2 days the expression of *neurog1* in the anteroventral domain of the otic placode [31], [32]. Therefore, the specification of otic neurons as judged by the induction of *neurog1* precedes hair cell specification. Moreover, hair cells from the utricle and saccule derive from a *neurog1*-positive domain in mice [43]. In chick, a clonal relation between sensory neurons and utricular epithelial cells was also found [44]. Together with a clonal relationship between neurons and hair cells, mutual antagonism between *atoh1* and *neurog1* has been shown [43], [45], [46]. In *Neurog1* ^-/-^ mouse embryos expansion of hair cells in the future utricle was observed, conversely increased number of neuronal cells was detected in *Atoh1* mutants [43].

We show that zebrafish *atoh1b* and *atoh1a* are induced before *neurog1*. Whether this discrepancy has any functional relevance is still not known. Disruption of *neurog1* by MO injection caused an expansion of hair cells from the posterior macula [35]. This was further confirmed in the present study, since *neurog1* mutants also display an increase on the expression of *her4* only in the posterior macula.

However, we also explored *neurog1* expression after blockade of *atoh1b*, as the first proneural gene defining the prosensory domain. Our data suggests that loss of *atoh1b* proneural activity does not modify *neurog1* expression, suggesting that the definition of the neurogenic domain is not influenced by *atoh1b* proneural gene. However, since two *atoh1* genes are present in zebrafish, further work deleting both *atoh1b* and *atoh1a* genes should provide better insights into proneural cross-regulation between sensory and neurogenic fates.

**Figure 7:**
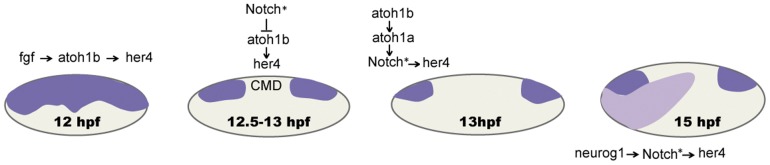
Sequential steps in *her4* activation and its regulation during sensori- and neurogenesis. (**A**) In the sensory domain *her4* expression is detected at 12 hpf in a broad medial territory of the otic placode and, at this early stage, it requires *atoh1b* and FGF signalling. (**B**) By 13 hpf *her4* gets restricted to the future anterior and posterior sensory domains being down-regulated in the CMD by Notch activity. (**C**) At later stages, *her4* expression requires Notch in order to be maintained in the future maculae. (**D**) In the neurogenic domain *neurog1* activates Notch, which in turn activates *her4* during lateral inhibition. Notch*: Notch active. *her4* expression in dark (sensory domain) and light (neurogenic domain) violet.

In conclusion, we show here that both, *neurog1* and *atoh1b* proneural genes, are main regulators of *her4* in the neurogenic and sensory domains. In the first, *her4* is involved in lateral inhibition to regulate the balance between neuronal precursors and differentiating neuronal cells, whereas in the latter, *her4* alone cannot regulate the number of hair cells during sensorigenesis, most probably helped by *her6*. Furthermore, in the sensory domain, Notch only acts upstream of *her4* at late stages but initial *her4* expression is downstream of *atoh1b*. Instead, at the neurogenic domain, the genetic cascade differs and *neurog1* first activates Notch, which in turn activates *her4* (Figure 7).

## Supporting Information

Figure S1 ***Her6*** **is expressed in similar domains than** ***her4. Her6*** **expression is restricted to the anterior and posterior sensory domains from its onset and is not induced at the CMD.**
(TIF)

## References

[1] Jarman AP, Grau Y, Jan LY, Jan YN (1993) atonal is a proneural gene that directs chordotonal organ formation in the Drosophila peripheral nervous system. Cell 73: 1307–1321. 8324823 10.1016/0092-8674(93)90358-w

[2] Jarman AP, Sun Y, Jan LY, Jan YN (1995) Role of the proneural gene, atonal, in formation of Drosophila chordotonal organs and photoreceptors. Development 121: 2019–2030. 7635049 10.1242/dev.121.7.2019

[3] Shailam R, Lanford PJ, Dolinsky CM, Norton CR, Gridley T, et al (1999) Expression of proneural and neurogenic genes in the embryonic mammalian vestibular system. J Neurocytol 28: 809–819. 10900086 10.1023/a:1007009803095

[4] Bermingham NA, Hassan BA, Price SD, Vollrath MA, Ben-Arie N, et al (1999) Math1: an essential gene for the generation of inner ear hair cells. Science 284: 1837–1841. 10364557 10.1126/science.284.5421.1837

[5] Zheng JL, Gao WQ (2000) Overexpression of Math1 induces robust production of extra hair cells in postnatal rat inner ears. Nat Neurosci 3: 580–586 10.1038/75753 10816314

[6] Woods C, Montcouquiol M, Kelley MW (2004) Math1 regulates development of the sensory epithelium in the mammalian cochlea. Nat Neurosci 7: 1310–1318 10.1038/nn1349 15543141

[7] Millimaki BB, Sweet EM, Dhason MS, Riley BB (2007) Zebrafish atoh1 genes: classic proneural activity in the inner ear and regulation by Fgf and Notch. Development 134: 295–305 10.1242/dev.02734 17166920

[8] Haddon C, Smithers L, Schneider-Maunoury S, Coche T, Henrique D, et al (1998) Multiple delta genes and lateral inhibition in zebrafish primary neurogenesis. Development 125: 359–370. 9425132 10.1242/dev.125.3.359

[9] Adam J, Myat A, Le Roux I, Eddison M, Henrique D, et al (1998) Cell fate choices and the expression of Notch, Delta and Serrate homologues in the chick inner ear: parallels with Drosophila sense-organ development. Development 125: 4645–4654. 9806914 10.1242/dev.125.23.4645

[10] Lanford PJ, Lan Y, Jiang R, Lindsell C, Weinmaster G, et al (1999) Notch signalling pathway mediates hair cell development in mammalian cochlea. Nat Genet 21: 289–292 10.1038/6804 10080181

[11] Ma Q, Chen Z, del Barco Barrantes I, de la Pompa JL, Anderson DJ (1998) Neurogenin1 is Essential for the Determination of Neuronal Precursors for Proximal Cranial Sensory Ganglia. Neuron 20: 469–482. 9539122 10.1016/s0896-6273(00)80988-5

[12] Andermann P, Ungos J, Raible DW (2002) Neurogenin1 defines zebrafish cranial sensory ganglia precursors. Dev Biol 251: 45–58. 12413897 10.1006/dbio.2002.0820

[13] Abello G, Khatri S, Giraldez F, Alsina B (2007) Early regionalization of the otic placode and its regulation by the Notch signaling pathway. Mech Dev 124: 631–645 10.1016/j.mod.2007.04.002 17532192

[14] Daudet N, Ariza-McNaughton L, Lewis J (2007) Notch signalling is needed to maintain, but not to initiate, the formation of prosensory patches in the chick inner ear. Development 134: 2369–2378 10.1242/dev.001842 17537801

[15] Murata J, Tokunaga A, Okano H, Kubo T (2006) Mapping of notch activation during cochlear development in mice: implications for determination of prosensory domain and cell fate diversification. J Comp Neurol 497: 502–518 10.1002/cne.20997 16736472

[16] Zine A, Aubert A, Qiu J, Therianos S, Guillemot F, et al (2001) Hes1 and Hes5 activities are required for the normal development of the hair cells in the mammalian inner ear. J Neurosci 21: 4712–4720. 11425898 10.1523/JNEUROSCI.21-13-04712.2001 PMC6762342

[17] Hartman BH, Basak O, Nelson BR, Taylor V, Bermingham-McDonogh O, et al (2009) Hes5 expression in the postnatal and adult mouse inner ear and the drug-damaged cochlea. J Assoc Res Otolaryngol 10: 321–340 10.1007/s10162-009-0162-2 19373512 PMC2757554

[18] Tateya T, Imayoshi I, Tateya I, Ito J, Kageyama R (2011) Cooperative functions of Hes/Hey genes in auditory hair cell and supporting cell development. Dev Biol 352: 329–340 10.1016/j.ydbio.2011.01.038 21300049

[19] Takke C, Dornseifer P, v Weizsacker E, Campos-Ortega JA (1999) her4, a zebrafish homologue of the Drosophila neurogenic gene E(spl), is a target of NOTCH signalling. Development 126: 1811–1821. 10101116 10.1242/dev.126.9.1811

[20] Kroehne V, Freudenreich D, Hans S, Kaslin J, Brand M (2011) Regeneration of the adult zebrafish brain from neurogenic radial glia-type progenitors. Development 138: 4831–4841 10.1242/dev.072587 22007133

[21] So JH, Chun HS, Bae YK, Kim HS, Park YM, et al (2009) Her4 is necessary for establishing peripheral projections of the trigeminal ganglia in zebrafish. Biochem Biophys Res Commun 379: 22–26 10.1016/j.bbrc.2008.11.149 19084503

[22] Yeo SY, Kim M, Kim HS, Huh TL, Chitnis AB (2007) Fluorescent protein expression driven by her4 regulatory elements reveals the spatiotemporal pattern of Notch signaling in the nervous system of zebrafish embryos. Dev Biol 301: 555–567 10.1016/j.ydbio.2006.10.020 17134690

[23] Itoh M, Kim CH, Palardy G, Oda T, Jiang YJ, et al (2003) Mind bomb is a ubiquitin ligase that is essential for efficient activation of Notch signaling by Delta. Dev Cell 4: 67–82. 12530964 10.1016/s1534-5807(02)00409-4

[24] Golling G, Amsterdam A, Sun Z, Antonelli M, Maldonado E, et al (2002) Insertional mutagenesis in zebrafish rapidly identifies genes essential for early vertebrate development. Nat Genet 31: 135–140 10.1038/ng896 12006978

[25] Madelaine R, Blader P (2011) A cluster of non-redundant Ngn1 binding sites is required for regulation of deltaA expression in zebrafish. Dev Biol 350: 198–207 10.1016/j.ydbio.2010.10.025 21034732

[26] Kimmel CB, Ballard WW, Kimmel SR, Ullmann B, Schilling TF (1995) Stages of embryonic development of the zebrafish. Dev Dyn 203: 253–310 Available: http://www.ncbi.nlm.nih.gov/pubmed/8589427. 8589427 10.1002/aja.1002030302

[27] Thisse B, Heyer V, Lux A, Alunni V, Degrave A, et al (2004) Spatial and temporal expression of the zebrafish genome by large-scale in situ hybridization screening. Methods Cell Biol 77: 505–519. 15602929 10.1016/s0091-679x(04)77027-2

[28] Itoh M, Chitnis AB (2001) Expression of proneural and neurogenic genes in the zebrafish lateral line primordium correlates with selection of hair cell fate in neuromasts. Mech Dev 102: 263–266. 11287207 10.1016/s0925-4773(01)00308-2

[29] Gajewski M, Elmasri H, Girschick M, Sieger D, Winkler C (2006) Comparative analysis of her genes during fish somitogenesis suggests a mouse/chick-like mode of oscillation in medaka. Dev Genes Evol 216: 315–332 10.1007/s00427-006-0059-6 16544152

[30] Radosevic M, Robert-Moreno A, Coolen M, Bally-Cuif L, Alsina B (2011) Her9 represses neurogenic fate downstream of Tbx1 and retinoic acid signaling in the inner ear. Development 138: 397–408 10.1242/dev.056093 21205785

[31] Pujades C, Kamaid A, Alsina B, Giraldez F (2006) BMP-signaling regulates the generation of hair-cells. Dev Biol 292: 55–67 10.1016/j.ydbio.2006.01.001 16458882

[32] Alsina B, Abello G, Ulloa E, Henrique D, Pujades C, et al (2004) FGF signaling is required for determination of otic neuroblasts in the chick embryo. Dev Biol 267: 119–134 10.1016/j.ydbio.2003.11.012 14975721

[33] Bae YK, Shimizu T, Hibi M (2005) Patterning of proneuronal and inter-proneuronal domains by hairy- and enhancer of split-related genes in zebrafish neuroectoderm. Development 132: 1375–1385 10.1242/dev.01710 15716337

[34] Scholpp S, Delogu A, Gilthorpe J, Peukert D, Schindler S, et al (2009) Her6 regulates the neurogenetic gradient and neuronal identity in the thalamus. Proc Natl Acad Sci U S A 106: 19895–19900 10.1073/pnas.0910894106 19903880 PMC2775703

[35] Sapede D, Dyballa S, Pujades C (2012) Cell lineage analysis reveals three different progenitor pools for neurosensory elements in the otic vesicle. J Neurosci 32: 16424–16434 10.1523/JNEUROSCI.3686-12.2012 23152625 PMC6794044

[36] Chitnis AB (1995) The role of Notch in lateral inhibition and cell fate specification. Mol Cell Neurosci 6: 311–321. 8742272

[37] Lewis J (1998) Notch signalling and the control of cell fate choices in vertebrates. Semin Cell Dev Biol 9: 583–589 10.1006/scdb.1998.0266 9892564

[38] Takebayashi K, Akazawa C, Nakanishi S, Kageyama R (1995) Structure and promoter analysis of the gene encoding the mouse helix-loop-helix factor HES-5. Identification of the neural precursor cell-specific promoter element. J Biol Chem 270: 1342–1349. 7836401 10.1074/jbc.270.3.1342

[39] Chrysostomou E, Gale JE, Daudet N (2012) Delta-like 1 and lateral inhibition during hair cell formation in the chicken inner ear: evidence against cis-inhibition. Development 139: 3764–3774 10.1242/dev.074476 22991441

[40] Kiernan AE, Cordes R, Kopan R, Gossler A, Gridley T (2005) The Notch ligands DLL1 and JAG2 act synergistically to regulate hair cell development in the mammalian inner ear. Development 132: 4353–4362 10.1242/dev.02002 16141228

[41] Brooker R, Hozumi K, Lewis J (2006) Notch ligands with contrasting functions: Jagged1 and Delta1 in the mouse inner ear. Development 133: 1277–1286 10.1242/dev.02284 16495313

[42] Takebayashi S, Yamamoto N, Yabe D, Fukuda H, Kojima K, et al (2007) Multiple roles of Notch signaling in cochlear development. Dev Biol 307: 165–178 10.1016/j.ydbio.2007.04.035 17531970

[43] Raft S, Koundakjian EJ, Quinones H, Jayasena CS, Goodrich L V, et al (2007) Cross-regulation of Ngn1 and Math1 coordinates the production of neurons and sensory hair cells during inner ear development. Development 134: 4405–4415 10.1242/dev.009118 18039969

[44] Satoh T, Fekete DM (2005) Clonal analysis of the relationships between mechanosensory cells and the neurons that innervate them in the chicken ear. Development 132: 1687–1697 10.1242/dev.01730 15743876

[45] Matei V, Pauley S, Kaing S, Rowitch D, Beisel KW, et al (2005) Smaller inner ear sensory epithelia in Neurog 1 null mice are related to earlier hair cell cycle exit. Dev Dyn 234: 633–650 10.1002/dvdy.20551 16145671 PMC1343505

[46] Jahan I, Pan N, Kersigo J, Calisto LE, Morris KA, et al (2012) Expression of Neurog1 instead of Atoh1 can partially rescue organ of Corti cell survival. PLoS One 7: e30853 10.1371/journal.pone.0030853 22292060 PMC3265522

