# Supplementary figures and images for: The Role of her4 in Inner Ear Development and Its Relationship with Proneural Genes and Notch Signalling
Source: PLoS One. 2014 Oct 9;9(10):e109860. doi: 10.1371/journal.pone.0109860 (PMC4192589; doi:10.1371/journal.pone.0109860)

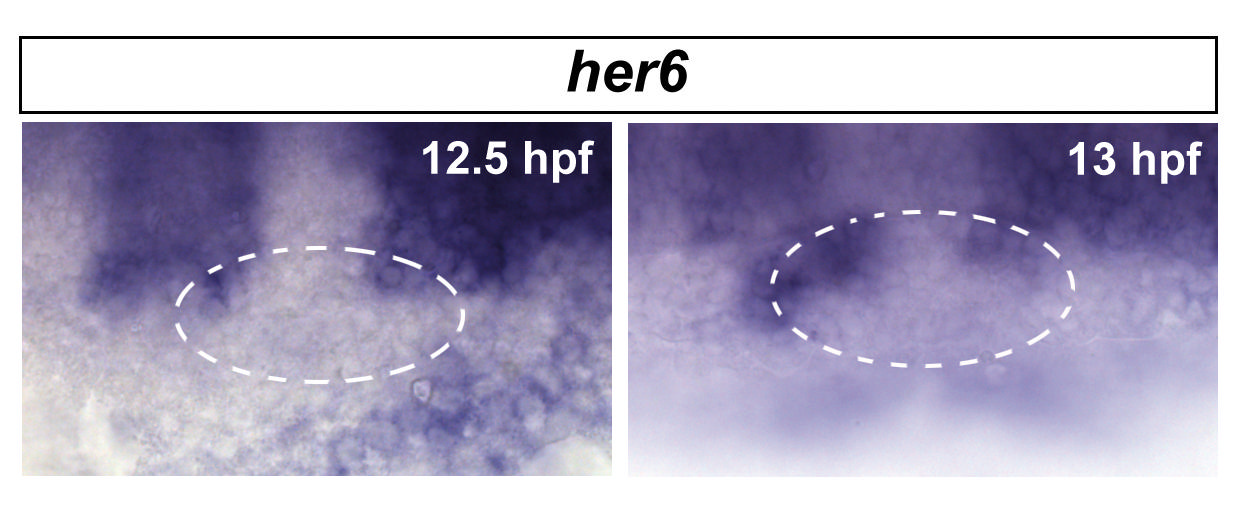

Supplement: Figure S1 — Her6 is expressed in similar domains than her4. Her6 expression is restricted to the anterior and posterior sensory domains from its onset and is not induced at the CMD. (TIF) [file pone.0109860.s001.tif]
